# Jakyakgamcho-tang and Its major component, paeonia lactiflora, exhibit potent anti-glycation properties

**DOI:** 10.20463/jenb.2016.0049

**Published:** 2016-12-31

**Authors:** Junghyun Kim, Chan-Sik Kim, Young Sook Kim, IK Soo Lee, Jin Sook Kim

**Affiliations:** 1 Korean Medicine Convergence Research Division, Korea Institute of Oriental Medicine, Daejeon Republic of Korea

**Keywords:** Advanced glycation end products, Jakyakgamcho-tang, Paeoniae Radix, Glycyrrhizae Radix

## Abstract

**[Purpose]:**

Advanced glycation end products (AGEs) have been implicated in the pathogenesis of diabetes and other age-related diseases. AGE inhibitors or breakers, such as aminoguanidine and alagebrium, have been proposed as therapeutic agents for AGE-related disorders. Jakyakgamcho-tang (JGT) is a well-known traditional herbal formula, which consists of the radix of *Paeonia lactiflora* Pallas (PR) and the radix and rhizome of *Glycyrrhiza uralensis* Fisch (GR). The purpose of this study was to evaluate the inhibitory and breaking activities of JGT, PR, and GR against AGEs.

**[Methods]:**

JGT, PR, and GR extracts were prepared in hot water. We performed in vitro assays to evaluate their inhibitory activity against glycation of bovine serum albumin (BSA) by high glucose and their ability to break the already formed AGEs.

**[Results]:**

In the *in vitro* AGE formation assay, JGT and PR dose-dependently inhibited AGE-BSA formation (half-maximal inhibitory concentration, IC_50_, = 41.41 ± 0.36 and 6.84 ± 0.09 μg/mL, respectively). In the breakdown assay of the preformed AGE-BSA-collagen complexes, JGT and PR exhibited potent breaking activities (IC_50_ = 6.72 ± 1.86 and 7.45 ± 0.47 μg/mL, respectively). However, GR showed a weaker inhibitory activity and no breaking activity against AGEs.

**[Conclusion]:**

This study suggests that JGT and PR could be valuable drug candidates for treatment of AGE-related diseases by reducing AGE burden.

## INTRODUCTION

Glycation process is a spontaneous non-enzymatic reaction between the free reducing sugars and free amino groups of proteins, DNA, and lipids to form an Amadori product. The Amadori product undergoes further complex reactions, such as dehydration and rearrangement, resulting in the formation of irreversible products, known as advanced glycation end products (AGEs). AGEs involve heterogeneous sugar-derived irreversible protein modifications that have been implicated in the pathogenesis of diabetes and other age-related diseases^1^. AGEs can accumulate in the intracellular spaces and covalently cross-link with proteins, particularly collagen. In addition to the circulating proteins, collagen and other matrix proteins that have very slow turnover rates are expected to be glycated and eventually transformed into AGEs in diabetes and aging process^2, 3^.

It has been suggested that inhibition of the glycation process can suppress the progress of several AGE-related disorders. Aminoguanidine, a nucleophilic hydrazine compound, can inhibit the glycation reaction *in vitro* and *in vivo*^4, 5^. The mechanism of action of aminoguanidine may involve trapping of the dicarbonyl metabolites, such as methylglyoxal^6^. In several animal experiments, aminoguanidine inhibited AGE formation and prevented the renal, retinal, neural, and vascular complications of diabetes^7^. However, aminoguanidine was not clinically used to treat diabetic complications because of its adverse effects, such as pro-oxidant activities^8^ and inhibition of NO synthase^9^.

Vasan *et al*. developed a new compound, N-phenacylthiazolium (PTB), which was designed to break the pre-existing AGE cross-links in tissue proteins^10^. PTB was found to break the cross-links between diabetic rat tail tendon collagen and AGEs *in vitro*. However, PTB is unstable in physiological buffers^11^. Alagebrium, a more stable derivative of PTB, was developed. It could reverse cardiovascular dysfunction mediated by AGE cross-links^12-14^. However, clinical trials on alagebrium were not finished owing to financial constraints^15^.

Some natural and synthetic compounds have been proposed as AGE inhibitors^16^. Generally, botanical products are often considered safer than synthetic compounds. Therefore, the interest in the use of herbal products has been increasing^17^. Jakyakgamcho-tang (JGT; Shaoyao-gancao-tang in Chinese; Shakuyaku-kanzo-to in Japanese) is a well-known traditional herbal formula, which consists of the radix of Paeonia lactiflora Pallas (PR) and the radix and rhizome of *Glycyrrhiza uralensis* Fisch (GR). This herbal formula has been used for various indications, including analgesia and anti-spasms^18^. Despite the various effects of JGT, its inhibitory effects on the glycation process have not been investigated yet. Moreover, to the best of our knowledge, there are no previous studies on the breaking activity of JGT against the preformed AGEs. Therefore, the present study aimed to evaluate the inhibitory action of JGT on the formation of AGEs and AGEs cross-links with proteins *in vitro*. Furthermore, we compared its effectiveness with that of aminoguanidine and alagebrium.

## METHODS

### Preparation of JGT and HPLC analysis

PR and GR were purchased from Baekjedang herb store (Daejeon, Korea). Voucher specimens of PR and GR were deposited at the Herbarium of the Korea Institute of Oriental Medicine, Korea. For preparation of JGT, 100 g of PR and 50 g of GR were accurately weighed and mixed. Distilled water (900 mL) was added to the mixed herbs and extracted at 100 °C for 2 h using a heat-reflux extractor. The extracted solution was filtered and freezedried to give JGT extract powder (18 g). For preparation of PR and GR extracts, 100 g of both PR and GR were accurately weighed and extracted separately as described above for JGT. Each extracted solution was filtered and freeze-dried to give extract powders of PR (15.4 g) and GR (12 g).

The contents of the major compounds of JGT were determined by high-performance liquid chromatography (HPLC) analysis. HPLC analysis was performed using an Agilent 1200 HPLC instrument (Agilent Technologies, USA) equipped with a binary pump (G1312A), vacuum degasser (G1322A), auto-sampler (G1329A), column compartment (G1316A), and diode array detector (DAD, 1365B). A Luna C-18 analytical column (i.d., 4.6 mm × 250 mm; particle size, 5 μm, Phenomenex) was used. The mobile phase consisted of 0.1 % formic acid in water (A) and acetonitrile (B). The mobile phase gradient elution was programmed as follows: 0-25 min, 90-83 % A; 25-60 min, 83-81 % A; and 60-70 min, 81-60 % A. The flow rate of the mobile phase was set at 1 mL/min. The column temperature was maintained at 40 °C. The sample injection volume was 5 μL and the DAD detector wavelength was set at 230 nm.

### Inhibitory activity on AGE formation

Bovine serum albumin (BSA, 10 mg/mL, Sigma Chemicals, MO, USA) was incubated at 4 °C for 7 days with glucose (0.2 M) in phosphate buffer (50 mM, pH 7.4). All the reagent and samples were sterilized by filtration through 0.2-mm membrane filters. The reaction mixture was then mixed with JGT and PR. Aminoguanidine (Sigma Chemicals, MO, USA) was used as a positive inhibitor. The levels of AGEs were determined by measuring AGE-specific fluorescence using a spectrofluorometer (excitation wavelength at 370 nm and emission wavelength at 440 nm, Synergy HT, BIO-TEK, VT, USA). We calculated the 50% inhibitory concentration (IC_50_) for AGE formation.

### Breaking activity on preformed AGE-collagen complexes

The ability of JGT to break the preformed AGEs was evaluated using a previously described method10. Briefly, 1 μg of glycated BSA (AGE-BSA, MBL international, Woburn, MA, USA) was pre-incubated in collagen-coated 96-well plates for 24 h, and the collagen-AGE-BSA complexes were then incubated with JGT, PR, and GR. Alagebrium (Suchem pharma Co., Wenzhou, China) was used as a positive AGE breaker. Collagen-AGE-BSA cross-linking was detected using mouse anti-AGE primary antibody (6D12, Wako, Osaka, Japan), horseradish peroxidase-linked goat-anti mouse IgG secondary antibody, and H2O2 substrate containing 2,2’-azino-bis(3-ethylbenzothiazoline-6-sulfonic acid) (ABTS) chromogen. Breakdown levels were measured as the percentage decrease in optical density (OD = 410 nm). We calculated the IC_50_ (μg/ mL) as 50 % inhibition of collagen-AGE-BSA cross-linking.

### Statistical analysis

Results were analyzed using one-way analysis of variance (ANOVA) followed by Tukey’s multiple comparison test using GraphPad Prism 4.0 (GraphPad Software, CA, USA).

## RESULTS

### HPLC analysis of JGT

Paeoniflorin and glycyrrhizin are known as the major ingredients of JGT19. To assure the quality of JGT, HPLC was applied for quantitative analysis of paeoniflorin and glycyrrhizin in JGT. The contents of paeoniflorin and glycyrrhizin were 59.55 ± 0.39 and 12.13 ± 0.01 mg/g, respectively (Table 1).

**Table 1.** The contents of paeoniflorin and glycyrrhizin in JGT.

Compounds	Content (Mean ± SD, n = 3)
	mg/g
Paeoniflorin	59.55 ± 0.39
Glycyrrhizin	12.13 ± 0.01

### Inhibitory effects of JGT and PR on AGE formation in vitro

The inhibitory activity of JGT and its component, PR, on AGE-BSA formation was evaluated. As shown in Table 2, JGT and PR dose-dependently inhibited the formation of AGE-BSA (IC_50_ = 41.41 ± 0.36 and 6.84 ± 0.09 μg/mL, respectively). In addition, in our previous study, GR showed inhibitory activity on AGE formation (IC_50_ = 120.20 μg/mL)^20^. The inhibitory activities of JGT and PR were stronger than that of aminoguanidine (IC_50_ = 78.28 ± 4.24 μg/mL), whereas GR showed a relatively weak anti-AGE activity.

**Table 2.** Inhibitory effects of JGT and PR on AGE formation.

Part used	Concentration (μg/mL)	Inhibitory effect	
		Inhibitory effect (%)	IC_50_ (μg/mL)
JGT	10	13.68 ± 0.43	41.41 ± 0.36
	25	30.98 ± 0.90	
	50	59.95 ± 0.25	
PR	2.5	14.17 ± 1.34	6.84 ± 0.09
	5	37.72 ± 1.03	
	10	74.20 ± 0.88	
Aminoguanidine	55.5	44.53 ± 2.33	78.28 ± 4.24
	74	49.97 ± 0.59	
	111	56.37 ± 0.67	

Inhibitory effect was expressed as the mean ± S.D. of triplicate experiments. IC50 values were calculated from the dose-inhibition curve.

### Breaking effects of JGT, PR, and GR on AGE-collagen cross-links

The ability of various concentrations of JGT, PR, and GR to break the cross-links in the preformed AGE-BSA-collagen complexes was investigated (Table 3). JGT and PR dose-dependently destroyed the cross-links in the preformed AGE-BSA complexes with rat tail tendon collagen (IC_50_ = 6.72 ± 1.86 and 7.45 ± 0.47 μg/mL, respectively), and their inhibitory activities were stronger than those of alagebrium (IC_50_ = 17.76 ± 3.25 mg/mL). However, GR did not show a breaking effect on AGE cross-links with collagen.

**Table 3.** Breaking effects of JGT, PR, and GR on the preformed AGE cross-links.

Part used	Concentration		Breaking effect	
			AGE-collagen complex (%)	IC_50_
JGT	1	μg/mL	62.11 ± 5.14	6.72 ± 1.86 μg/mL
	5		52.03 ± 4.04	
	10		44.88 ± 3.20	
PR	1	μg/mL	74.57 ± 5.66	7.45 ± 0.47 μg/mL
	5		63.34 ± 3.57	
	10		36.17 ± 3.93	
GR	50	μg/mL	120.15 ± 6.12	
	100		117.69 ± 3.42	
	250		100.64 ± 9.17	
Alagebrium	50	mg/mL	61.61 ± 5.53	17.76 ± 3.25 mg/mL
	100		57.80 ± 3.58	
	200		45.76 ± 4.41	

Inhibitory effect was expressed as the mean ± S.D. of triplicate experiments. IC50 values were calculated from the dose-inhibition curve.

## DISCUSSION

Many previous studies have reported that AGE formation and accumulation in tissues play a crucial role in the pathogenic processes of diabetic complications^21^. Therefore, inhibition of AGE formation is a potential therapeutic strategy for diabetic complications. In the present study, JGT and PR dose-dependently inhibited the formation of AGE-BSA complexes. In addition, we previously reported that GR exhibited an inhibitory activity on AGE formation^20^. The inhibitory activities of JGT and PR were stronger than that of aminoguanidine. However, GR showed a relatively weak anti-AGE activity. Moreover, the ability of various concentrations of JGT, PR, and GR to break the cross-links in the preformed AGE-BSA-collagen complexes was investigated. JGT and PR dose-dependently destroyed the cross-links in the preformed AGE-BSA complexes with rat tail tendon collagen, and their inhibitory activities were stronger than that of alagebrium. However, GR did not show a breaking effect on AGE cross-links with collagen. Collectively, we showed that JGT could act as potent AGE inhibitor and breaker. The anti-glycation activities of JGT may be, in part, owing to its active component, PR.

The cytotoxic effects of AGEs in diabetes have been reported in many previous studies^1^. AGEs are toxic, immunogenic, and capable of triggering cellular injury responses after uptake by specific cellular receptors^22, 23^. Moreover, no enzyme can break AGEs in human body. Thus, inhibition of AGE formation or breakdown of the preformed AGEs is of increasing importance in diabetes and other age-related diseases. Aminoguanidine could suppress AGE formation and thus prevent diabetic nephropathy, retinopathy, and neuropathy in several animal experiments^7^. However, owing to safety concerns, aminoguanidine is not currently used^24^. Recently, several researchers have suggested that a certain agent can destroy AGE-derived protein cross-links. The first identified AGE breaker, PTB, was introduced in 1996. Because PTB is unstable *in vitro*, this compound was not clinically successful. Other compounds, including alagebrium^12^, LR20, LR23, LR90^25, 26^, and C36^27^, were developed as AGE breakers. AGE breakers could reverse AGE accumulation *in vivo*^28^. Moreover, alagebrium prevented the accumulation of AGEs in the blood vessels^29^ and heart^30^. Because the clinical studies on these compounds were terminated or still under development, none of these compounds is clinically used. Thus, the search for anti-glycation drug candidates with high efficacy and safety pursues.

Medicinal herbs are rich sources of potential preventive and therapeutic agents. JGT is a well-known traditional herbal formula with an excellent safety profile^31^. The detailed mechanism of action of JGT as an AGE inhibitor and breaker is still not clear. Aminoguanidine suppresses AGE formation via interaction with the reactive dicarbonyl species and acting as dicarbonyl scavenger^6, 32^. Bang *et al*. showed that PR exhibited a radical-scavenging activity^33^. It is suggested that the radical-scavenging activity of PR could contribute to the inhibition of AGE formation. In addition, one of the major ingredients of PR is (-)-epicatechin^34^. Our previous study revealed that (-)-epicatechin exhibited a breaking activity on the preformed AGEs *in vitro* and *in vivo*. Generally, AGEs are mostly responsible for protein-protein cross-linking *in vivo*. The side chains attached to the pyrrole ring carbons in AGEs are susceptible to nucleophilic attack^22^. Because the C6 and C8 of the A-ring of (-)-epicatechin are nucleophilic^23^, (-)-epicatechin can attack the AGE cross-links. It is suggested that the breaking activity of JGT might be attributed to its bioactive ingredient, (-)-epicatechin.

In traditional Korean medicine, PR has been used to nourish the blood, regulate menses, and alleviate pain. GR has been used to stop cough and detoxify several toxic substances, whereas JGT has been used to treat muscle cramps. It was reported that this pharmacological effect of JGT was only observed when GR was mixed with PR^35^. Although GR exhibited a weaker inhibitory activity and no breaking activity on AGEs, the breaking activity of JGT was stronger than that of PR and GR. These results suggest that the activity of JGT may be owing to synergistic effects between its two components, PR and GR.

In conclusion, our study showed that JGT could act as AGE inhibitor and AGE cross-link breaker. These activities of JGT were largely attributed to its bioactive component, PR. These results suggest the potential utility of JGT and PR as AGE inhibitors and breakers to treat AGE-related diseases.
